# Survival of patients with stomach adenocarcinoma in North of Iran 

**Published:** 2014

**Authors:** Jamshid Yazdani-Charati, Ghasem Janbabaei, Siavosh Etemadinejad, Samaneh Sadeghi, Firoozeh Haghighi

**Affiliations:** 1 *Department of Biostatistics, Faculty of Health, Health Sciences Research Center, Mazandaran University of Medical Sciences, Sari, Iran*; 2 *Department of Internal Medicine, Faculty of Medicine, Mazandaran University of Medical Sciences, Sari, Iran*; 3 *Department of Occupational Health, Faculty of Health, Health Sciences Research Center, Mazandaran University of Medical Sciences, Sari, Iran*; 4 *Department of Biostatistics, Faculty of Health, Mazandaran University of Medical Sciences, Sari, Iran*; 5 *Department of Mathematics, Statistics and Computer Sciences, Tehran University, Tehran, Iran*

**Keywords:** Cox hazard proportional model, Life table, Parametric model, Stomach adenocarcinoma

## Abstract

**Aim:** This study was proposed for estimation of survival time in patients with stomach adenocarcinoma.

**Background:** North of Iran has a high mortality rate of stomach adenocarcinoma.

**Patients and methods:** The study was historical cohort. The samples were the patients with stomach adenocarcinoma referred to Tooba Clinic between three years (2007-2010). Survival estimates were calculated using the Kaplan-Meier method the effects of covariates on survival time were assessed by, using survival parametric regression model with gamma frailty.

**Results:** The survival probability of more than two years of patients was calculated 27.7% by using Kaplan Mayer method. The stage, metastasis, surgery, and age were the variables which affect the survival probability of patients, by using survival parametric regression model with gamma frailty and hazard ratio of patients with three treatment protocol was 0.43 times of others (P<0.01) and increasing of patients ages decrease life time of them significantly as per year increasing patient age, risk of death increased by 4% (P<0.04) and patients with staging disease lower 4 had hazard ratio lower than 0.46 times of stage 4 (P<0.01).

**Conclusion:** The survival time of our patients is much lower than the developed countries, which are related to latency in diagnosis and therapeutic limitations.

## Introduction

 Determination of survival time of patients with cancers is one of the most useful methods in management of cancer programs and evaluation of the therapeutic methods (1). However the survival time of the individuals in each statistical society is a random variable and only can be predicted with statistical methods. Survival time is the estimation of the time of the patients from the diagnosis to the time of death.

The estimation of patient's survival time, in the chronic diseases and cancer is one of the key points for physicians in treatment of patients.

Stomach adenocarcinoma is the fourth prevalent cancer and the second cause of death due to cancers (2). The mortality of the stomach adenocarcinoma is increasing among the world (3). This increase has been notable among the past 50 years and is more than the cardiovascular disease (4). This cancer has the high prevalence in Japan, western parts of Latin America, some parts of Caribbean countries and Eastern Europe, moderate prevalence in Finland, Austria and low prevalence in US, Australia and New Zealand (5).

Although the prevalence of the disease has been decreased in some countries, the definite reason of this decrease is unknown yet. The refrigeration of the foods, less use of soot foods, the promotion of nutrition of people and access of fresh fruits and vegetables are the probable reasons (6).

The most frequency is among 70 to 80 years old, although it may appear under age 20; the prevalence is rare under age 40 (7).

Cancers are the third cause of death in Iran (the cardiovascular disease are the first and the accidents are the second cause), so it comes to priorities of research (8).

The prevalence of stomach adenocarcinoma in men is more than women. The men-to-women ratio is 1.5 to 2 in 13 studied countries. However, the prevalence is equal under age 30 (9).

The probability of 5 years survival is about 21% in US. One of the important reasons of it is the diagnosis in advanced stages. The condition of the disease has not been assessed properly in Iran. However, it is one of the most prevalent cancers in Iran. The gastrointestinal cancers including stomach adenocarcinoma are very prevalent in Mazandaran. This study was proposed to survey the patient's survival and some of the affecting factors in referred patients to Tooba Clinic, Cancer Research Center of Mazandaran University of Medical sciences. 

## Patients and Methods

This study was a historical cohort. The samples were the patients with stomach adenocarcinoma referred to Tooba Clinic of Sari among 3 years (2007-2010). In their documents, call numbers and addresses, as well as other data such as age, gender, job, stage of disease, were gathered. Final information such as date of death, if happened, type of therapy (ies) including: Surgery, Radiotherapy and Chemotherapy gathered by next follow up. According to ICD-9 standard the patient with adenocarcinoma was an individual who his/her excised sample was diagnosed malignant by a pathologist. 

One hundred and ninety patients entered in the study. According to randomized refer of patients to the clinic, the time of patients refer follow Poisson distribution. Survival time of patients was considered from time of pathology diagnosis to dead time or end of the study.

At first the data were analyzed by descriptive statistics. The median of survival time of patients with stomach adenocarcinoma compared regarding age, gender, stage of disease and metastasis, using Kaplan Mayer method. Patient's survival curve compared with log rank test. Also, patient's survival probability was calculated by using life table. Then the effect of ancillary variables on patient's survival time was calculated using survival parametric regression model with gamma frailty. (α=0.05). SPSS and Stata software were used for analysis. 

## Results

In this research, 190 patients were studied. Fifty-eight individuals (30.5%) passed away, fifty-five individuals (28.9%) were alive and seventy-seven individuals (40.5%) were censored. One hundred and twenty nine (68.3%) persons were men and sixty (31.7%) were women. The mean age of the patients was 64 years (95%CI: 62.79-66.66). The mean age of men was 66 years (95%CI: 63.93-68.41), and of women was 61 years (95%CI: 57.80-65.24). The family history was positive for 20 patients (10.5%). Tumor was located in cardia in 20.6% of patients, body in 51.9% of patients and antrum in 27.5% of patients. Six percent of patients were in stage 1, 14.3% in stage 2, 31% in stage 3 and 48.2% in stage 4.

**Table 1:** Demographic and survival rate of patients in this research

**Stage**	**Average Survival Time (month)**	**Standard Error**	**95% Confidence Interval**	
			**Lower Bound**	**Upper Bound**
Stage I	28	2.81	22.5	33.5
Stage II	22.96	1.61	19.79	26.1
Stage III	20.92	1.75	17.48	24.36
Stage IV	12.95	1.66	9.69	16.21
Total	19.48	1.18	17.12	21.73
Percentages of survival times for gastric cancer patients in different intervals times				
				
Gender Survival time				
	Male (% alive)	Female (% alive)	Total (% alive)	Standard Error
6months	71	72	71	0.44
12months	58	61	59	0.68
18 months	54	51	54	0.90
30 months	28	-	18	0.54
Percentages of survival times for gastric cancer patients with respect to tumor situation and other characteristics				
				
Site of tumor N(%)				
	Cardio	Body		Antrum
	21(11.1)	53(51.9)		28(27.5)
Tumor background N(%)				
	Yes	No		Unknown
	10(5.3)	79(41.9)		92(48.9)
Genetic background N (%)				
	Yes	No		Unknown
	20(10.5)	79(41.9)		92(48.9)
Status N (%)				
	Alive	Censor		Dead
	55(28.9)	77(40.5)		58(30.5)

Eighty five point two percent of all patients were operated, in stage 4, 79% of patients were operated. Table 1 shows the demographic and survival rate of patients in various periods. One-year survival probability was 60.3% and 2 years survival probability was 27.7% by Kaplan Mayer method. Mean lifelong of patients was 19.95 months (95%CI: 17.77-22.13).

Using life table method, one year survival probability was 59% and more than 30-month survival probability was 18%. Mean of patient's life was 19 months. Two methods estimated the same results.

**Figure 1:**
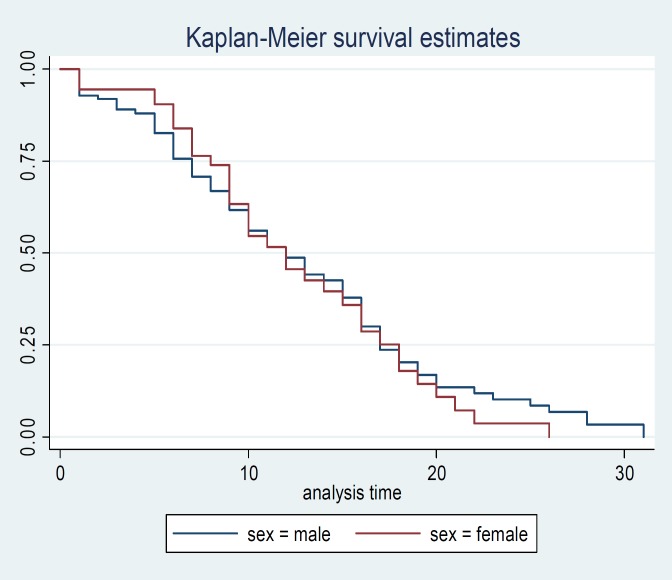
Survival time curve for gastric cancer patients in terms of gender in North of Iran

One-year survival probability in patients without surgery procedure was 59%, 17 months survival probability of them was 32% and mean of their survival was 12 months (95%CI: 7.93-16.30). These values for the patients whom operated were 63%, 57%, and 20%, 4 months (95%CI: 7.93-16.30).

Comparison of survival probability between two genders by log rank test did not show significant difference (P>0.05) (Figure 1). One year survival probability was estimated 58% for men and 61% for women, but the survival probability in women decreased more than men with time and 2-year survival probability reached 51% for men and 30.29 for women. Mean of life long was estimated 20.06 months (95%CI: 17.38-22.74) in men and 17.24 months (95%CI: 14.28-20.19) in women. Berslow and Taron ware tests confirmed the log rank test and did not show any significant difference between men and survival time. The mean of the survival time of patients without metastasis was 20.9 months (95%CI: 18.09-23.86) and patients with metastasis was 17.96 months (95%CI: 13.18-20.78), which declares that the metastasis is an effective factor in survival time of the patients.

**Table 2:** Average of survival times in month for gastric cancer patients in Mazandaran Province in terms of treatments

Treatment	Estimate of survival times (month)	95% C Confidence interval	
		Lower Bound	Upper Bound
CT	12.1	7.86	16.25
CT+ Surgery	17.6	14.13	20.96
CT+ RT+ Surgery	22.9	19.94	26.06

**Figure 2:**
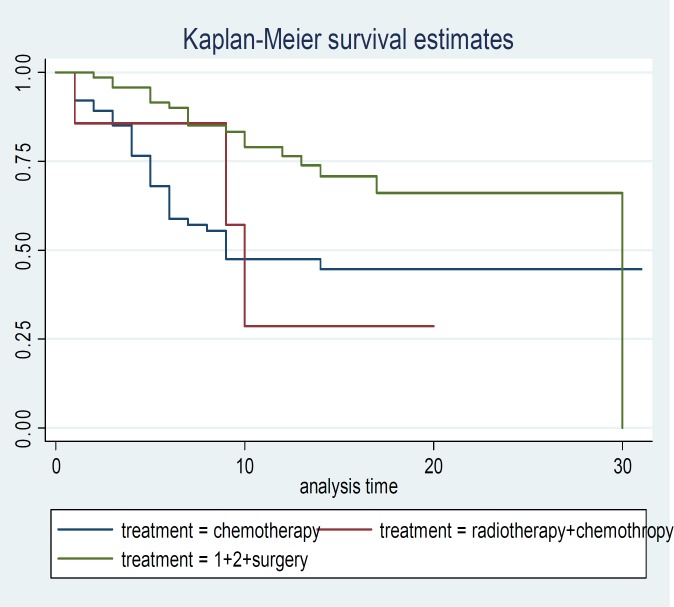
Survival time curve for gastric cancer patients in terms of treatments in Mazandaran province

The mean of survival time for patients was 28 months (95%CI: 22.5-23.5) in stage 1, 23.9 months (95%CI: 19.79-26.11) in stage 2, 20.9 months (95%CI: 17.48-24.36) in stage 3 and 12.9 months (95%CI: 9.69-16.20) in stage 4 of the cancer. There is a significant relationship between stage of the cancer and the survival time.

**Table 3:** MLE, Log-Likelihood, and AIC for data

Model	MLE	Log(Likelihood)	AIC
Weibull			
	α=2.61 β=17.68	-187.0347	378.0694
Gamma			
	α= 4.9 β=3.18	-187.5267	379.0534
Extension of exponential			
	-207.2298	-207.2298	418.4596

The patients which received 3 treatment protocol including surgery, chemotherapy and radiotherapy had the higher survival time than the patients received one of them (Table 2 and Figure 2). The protocol of the treatment was effective on patient's survival time. Log rank test also showed that this difference is significant. Figure 2 shows this difference.

In the next phase we considered all of the variables using survival parametric regression model with gamma frailty to evaluate the effects of them on patient's survival time. It showed that the stage of the cancer, metastasis, surgery and age are effective variables on patient's survival time, but the gender, job and positive family history do not affect survival time.

We were interested to fit a parametric model on the data. Empirical hazard function shows the progressive hazard. We consider Weibul, distribution as final model (10). The fitness of these models on the data is shown in Table 3. As it is obvious the Weibul distribution has the most fitness due to ACI criteria. After fitting Weibul regression model with gamma frailty, the result of them was shown in Table 4.

 As it was shown in Table 4, hazard ratio of patients with 3 treatment protocol was 0.43 times of others (P<0.01) and increasing of patients ages decrease life time of them significantly as per year increasing patient age, risk of death increased by 4% (P<0.04) and patients with staging disease lower 4 had hazard ratio lower than 0.46 times of stage 4. (P<0.01) on the other hand, the frailty variance of this model was calculated 2.64 (P<0.01). 

## Discussion

The main goal of this study was assessing the survival time of the patients with stomach adenocarcinoma and its effective factors. It may help the medical and health system for their therapeutic and prophylactic programs. In the recent years the infectious diseases have been decreased due to improvement in health, so the cancers have more important role in mortality of Iranian society. Various reports mentioned that stomach adenocarcinoma is very prevalent in Iran. It is the second prevalent cancers in men and totally the fourth prevalent cancer in Iran. As it is diagnosed and treated lately, its mortality is very high (11-13).

The north and northwest parts of the country are considered as high-risk regions for the stomach adenocarcinoma. A space cluster of the stomach cancer is described for Mazandaran and Golestan Provinces, located in the bank of the Caspian sea (14). Other studies, such as Hajian study in radiotherapy center of Babolsar (15), also showed the high prevalence of the disease in Mazandaran. 

Although the prevalence of the stomach cancer is decreasing in developed countries, it is increasing in developing countries. The putative causes are the aging of the population, nutritional problems, smoking and alcohol abuse (16).

The cancer is common in fourth decade of life and its prevalence increases by age. The weakness of immune system has a role in this increase (7).

The patient survival probability is an important issue in research of cancers and there are numerous studies in the various countries. Five years survival of the patients with stomach adenocarcinoma after the surgery is reported 29.6% in China, 4.4% in Thailand, 37% in US, 22% in Switzerland and 30% in France (17-21).

The lowest prevalence in Iran is reported from Khoozestan and Bakhtiari Provinces, and the highest prevalence from Ardabil Province with ARs (adjusted rate) of 49.1 in men and 25.4 in women. The mean survival time is 9.6 months in Ardabil (22-27).

The study made by Falah M. (from TAMIPERE University) in 5 provinces: Ardabil, Gilan, Mazandaran, Golestan and Kerman showed that the most frequent cancer among men was stomach adenocarcinoma (prevalent rate: 25). In women the most frequent cancer was the breast cancer (prevalent rate: 13.3) and the stomach adenocarcinoma was the second (prevalent rate: 9.3) (24). One-year survival time is reported 40% in Europe (15) and 54.23% in China (28).

**Table 4:** The results of Weibull model parametric along with gamma frailty in the gastric cancer patient’s survival multivariate analysis

	Hazard ratio	Standard error	P-value	95% confidence interval
Age	1.04	0.02	0.04	1.00-1.08
Three treatment protocol	0.43	0.13	<0.0001	0.24-0.76
Disease stage < 4	0.46	0.11	<0.0001	0.26-0.69
Frailty variance	2.64	1.33	0.001	0.99-7.08

Two-year survival probability of our samples was 27.7%, which is less than other countries such as US, Switzerland, France and China (16, 20, and 18). It seems to be related to the latency of patient's diagnosis that leads to lower efficiency of the treatments. The survival probability and median of survival time of men was more than women, however, it was not statistically significant. This finding is compatible with the studies made in other countries, which revealed that the lifetime of men and women do not show significant difference (27-30).

Unfortunately, 5-year survival probability was zero in our study.

Thirty six percent of patients had metastasis and lifetime of these patients was much lower than the others. Metastasis happens in advanced stages of cancer and causes the reduction of survival probability. This finding has been confirmed in other researches (17,19,25,29,31,32).

Survival parametric regression model with gamma frailty showed that increasing the patients age significantly decreases survival. Risk of death increased by 4%, for each year increment of age (*P*<0.05). This also has been found in the researches in US (33), Japan, and Italy (34). 

Stage of cancer significantly affects the patient's lifetime. Two years survival probability of patients was 28 months in stage 2 and 12 months in stage 4 of the cancer. As 48.2% of the patients came at stage 4, the survival probability estimated low in total patients.

Sixty eight point nine percent of patients in Thailand came at stage 4 and their 5 years survival probability was low (4.4%). Eighty two percent of patients in Malaysia came at stage 4. Therefore, 16% of them were operable (18,35). The effect of stage of cancer is seen in developed countries too (17,26,30).

Multivariate analysis showed that the age, stage of cancer and treatment protocol had the significant effect on patients’ life time, but the gender, metastasis and location of cancer did not affect significantly. These finding was confirmed with researches of Japan (31-32) and Switzerland (20) but in US studies the tumor location had effect as well as other variables (26). Mazandaran is located adjacent to the Alborz Mountains and the coalmines. The water is hard and contains high amounts of minerals. As well as these risk factors, the regiment must be considered (36).

Survival probability of patients with stomach adenocarcinoma is low in Mazandaran, which may be related to the lack of diagnostic facilities and the comprehensive strategic program for control in the province. Therefore, we recommend improving the diagnostic facilities, education of people and a comprehensive program for control of stomach adenocarcinoma.
